# Seasonal variation of acute appendicitis

**DOI:** 10.12669/pjms.343.14793

**Published:** 2018

**Authors:** Waqas Ahmed, Muhammad Saeed Akhtar, Shahum Khan

**Affiliations:** 1 Dr. Waqas Ahmed, MBBS, FCPS. Department of Surgery, Combined Military Hospital, Kohat, Pakistan; 2 Dr. Muhammad Saeed Akhtar, MBBS, MACS, FCPS. Department of Surgery, Combined Military Hospital, Kohat, Pakistan; 3 Dr. Shahum khan, MBBS. Department of Surgery, Combined Military Hospital, Kharian, Pakistan

**Keywords:** Acute appendicitis, Appendectomy, Diagnosis, Variation

## Abstract

**Objective:**

To determine the seasonal variation of acute appendicitis.

**Methods:**

A total of 320 patients fulfilling the inclusion criteria were enrolled in the study after getting the written informed consent. Appendectomies were performed by consultant surgeons and residents. After surgery histopathological examination of specimens was performed by consultant Histopathologists at Armed Forces Institute of Pathology Rawalpindi and CMH Peshawar. The patients presenting in different four seasons September to November as autumn, December to February as winter, March to May as spring, June to August as summer, were compared to determine seasonal variations.

**Results:**

In our study out of all 320 patients 188 (58.75%) were males and 132 (41.25%) were females. Sixty patients (18.75 %) presented in autumn season (Sep-Nov), 52 patients (16.25%) in winter season (Dec-Feb), 78 (24.25%) patients in Spring season (Mar-May).130 (40.62%) patients presented in Summer season (Jun-Aug). There was almost 24.37% increased incidence in summer as compared to winter season, 21.87% increased incidence as compared to autumn season, 16.37% increased incidence as compared to spring season.

**Conclusion:**

Acute appendicitis incidence is increased in summer months in Pakistan. Preventive measures can be taken during summer season (June to Aug) to decrease morbidity and mortality associated with this disease.

## INTRODUCTION

Acute appendicitis is the most common surgical emergency presenting in emergency department.^1^ worldwide Appendectomy is one of the most common operations.^2^ The prevalence of this condition is 28.6%.^3^ There is almost equal distribution amongst males and females.^4^ Moreover, the etiology of the appendicitis still remains unclear and multifactorial. The disease has been attributed to a variety of possible causes which include mechanical obstruction^5^, inadequate dietary fibre, smoking^6^, air pollution and familial susceptibility.^7^ Acute appendicitis presents throughout the year but incidence is increased in some particular months.^8^ There are many variations in the incidence of acute appendicitis. Various studies have been done to determine the seasonal variation of acute appendicitis but with variable results.^9^ Some studies have concluded increased incidence in summer months.^6^ The exact reason why acute appendicitis cases present in summer more than other seasons is still not clear. Various extrinsic factors such as air pollution^10^, gastrointestinal infection^11^ and low fiber diet during summer months could contribute to the higher incidence of appendicitis.^11^

Although numerous epidemiological studies on appendicitis have been conducted, most have focused on Western populations^12^, relatively few epidemiological studies have focused on appendicitis in Asian populations. Lee et al^13^ reported the epidemiological features and lifetime risk of appendicitis and appendectomy in South Korea. These studies were mainly concerned with the monthly variation in the incidence of acute appendicitis^14^ the volume-outcome relationship of acute appendicitis.^14^

There is very sparse research data regarding seasonal variation of acute appendicitis in Pakistan. The rationale of our study was to determine whether there is a seasonal variation regarding incidence of acute appendicitis. If a strong association found that will help in taking preventive measures to decrease incidence of this disease.

## METHODS

This study was conducted after the approval of hospital ethical committee at the department of general surgery Kohat, Pakistan, from Sep 2015 to Aug 2016. All patients (aged 10 to 50 years) who presented in the emergency department with right lower quadrant pain and underwent appendectomy were enrolled in the study. Patients having concomitant upper respiratory tract infections, generalized peritonitis, pregnant females or palpable appendicular mass on examination and the patients with CLD were excluded. Informed written consent was taken for participation in the study after explaining the objectives, benefits and drawbacks of the study. All patients received routine medical attention for acute appendicitis including detailed medical history, complete physical examination and required investigations.

Decision to operate upon was based on overall clinical diagnosis of the patients, Laboratory investigations and Ultrasound abdomen. Surgeries were performed by consultant surgeons and residents in surgery. Post operatively Specimens were sent to histopathology department Armed Forces Institute of Pathology (AFIP) and Combined Military Hospital Peshawar. Diagnosis of acute appendicitis was confirmed by histopathology .The period from September to November was defined as “Autumn season “. December to February was defined as “winter season”. March to May was defined “Spring season”. June to August was defined as “Summer season”. All the data was entered in table and seasonal variation was studied with males and females percentages.

## RESULTS

A total of 320 patients were included in the study during the study period from Sep 2015 to Aug 2016. Out of total 320 patients 188 (58.75%) patients were males and 132 (41.25%) were females. Male to female ratio was almost 1.5:1. Age distribution ranged from 10 – 50 years. Mean age was 24.25 years (SD = 5) Median 28 years. Majority of the patients belonged to second and third decades of life.

Statistical analysis of the study was done using Table-I to determine seasonal variation of acute appendicitis. In our study 320 cases were diagnosed as acute appendicitis on clinical examination and Post operatively confirmed by histopathology. Out of these 320 patients 60 patients (18.75 %) presented in autumn season from Sep to Nov 2016, 52 patients (16.25%) in winter season from Dec to Feb, 78 (24.25%) patients in spring season from Mar to May.130 (40.62%) patients presented in summer season ranging from Jun to Aug. There was almost 24.37% increased incidence in summer as compared to winter season, 21.87% increased incidence as compared to autumn season, 16.37% increased incidence as compared to spring season.

**Table-I:** Seasonal variation of acute appendicitis.

Duration	Total patients	Percentages	Males	Females
Sep – Nov(autumn)	60	18.75%	35	25
Dec – Feb (winter)	52	16.25%	28	24
Mar – May (spring)	78	24.25%	48	30
Jun – Aug(summer)	130	40.62%	77	53

Total	320	100%	188(58.75%)	132(41.25%)

## DISCUSSION

Acute appendicitis is the most common surgical emergency presenting in emergency department. The etiology of this disease is still poorly understood. The high prevalence of intestinal parasites and bacterial infections have been associated with some cases of acute appendicititis. Seasonal peaks of infections by various intestinal pathogens like salmonellosis, Escherichia coli, Entamoeba histolytica, Ascaris lumbricoides, Enterobius vermicularis and Strongyloides stercoralis which are associated in the pathogenesis of acute appendicitis exhibit a summer peak in some countries.^15^ Various studies have been done to determine the seasonal variation of acute appendicitis but with variable results.^16^ Studies in some warmer countries like United States, Canada, Iran, and South Africa showed peak incidence of AA during the warmer periods.^16^ It seems reasonable to assume that higher temperatures are associated with a higher risk of developing AA. Whether temperature plays a minor or major role in the development of AA when compared to other risk factors, or whether it is an independent risk factor at all, remains to be solved in further studies.

Our study conducted at Combined Military Hospital Kohat for a duration of one year to determine the seasonal variation of acute appendicitis. Out of 320 patients 130 patients 40.62% presented in Summer season ranging from June to August while 60 patients (18.75%) presented in Autumn season from Sep to Nov 2016, 52 patients (16.25%) in winter season from Dec to Feb, 78 (24.25%) patients in Spring season from Mar to May in Pakistan. Seasons had a statistically significant impact on the incidence of Acute Appendicitis with a relatively increased incidence in acute appendicitis in summer season.

Saps et al^17^ described the effect of seasonality on NSAP in children. The highest incidence of AA was found in summer and the lowest levels in winter. The exact reasons for increased incidence of AA during the warm period are not clear but various speculations have been made like, humidity, dehydration, diet effects, infections or changes in atmospheric pressure.

In a study conducted at Finland by Imre et al^18^ in various centres of Finland showed clear correlation of seasonality with acute appendicitis with increased incidence in Summer season with almost comparable results to our study.

A study was conducted in India by Babita et al^19^ over a decade (from January 2003-July 2012). A total of 395 cases were included. The cases were maximum in the month of August and minimum were noticed in January while in our study maximum incidence was seen in Summer Season ranging from June to Aug.^19^

Gallerani et al^20^ in their study confirmed seasonal variations of Acute appendicitis with almost comparable results with our study. This variation shows the possibility of various extrinsic factors such as dehydration, humidity, allergens, sun radiation, and viral and bacterial infections in the etiology of acute appendicitis.

Wei et al.^14^ analyzed the relationship between the incidence of appendicitis and climatic factors, including ambient temperature, relative humidity, atmospheric pressure, rainfall, and hours of sunshine; they reported a positive correlation between ambient temperature and the incidence of appendicitis. Kaplan et al reported a significant effect of air pollution on the incidence of appendicitis in the summer season.

In a study conducted in by Rai et al^12^ Clear seasonal variation was observed in the incidences of appendicitis, acute appendicitis, and appendectomy for both genders; the peak incidence seen in the summer season and decreased in the winter season. This pattern has been observed in several previous studies. In addition, the present study observed a slight but consistent increase in the incidence of perforated appendicitis in the summer season, which is inconsistent with several previous studies.^12^

Another study was conducted in Taiwan by Lin et al^21^ all in 2015. The highest incidence of appendicitis was found in persons aged 15 to 29 years; males had higher rates of appendicitis than females at all ages except for 70 years and older. Appendicitis rates were 11.76 % higher in the summer than in the winter months. Whereas in our study 24.37% higher incidence was seen in Summer than winters. Rest of the results were almost comparable.

### Limitation of our study

The study population was not a true representation of the society as most of the patients belonged to a particular age group, military background and region of Kohat that cannot be generalized all over Pakistan.

## CONCLUSION

Acute appendicitis incidence is increased in summer months in Pakistan. This seasonal variation is associated with various causes. Preventive measures can be taken during summer season from June to Aug to decrease morbidity and mortality associated with this disease.
